# The effect of voluntary head movements on postural kinetics in the standing cat

**DOI:** 10.7717/peerj.8186

**Published:** 2019-12-12

**Authors:** Yang Song, Meizi Wang, Julien Steven Baker, Yaodong Gu

**Affiliations:** 1 Faculty of Sports Science, Ningbo University, Ningbo, China; 2 Research Institute for Exercise and Health Sciences, Hong Kong Baptist University, Hong Kong, Hong Kong

**Keywords:** Postural control, voluntary head movement, Kinetics, Cat

## Abstract

**Background:**

Although the postural instability accompanying bilateral vestibular loss in human and quadrupeds during lateral head movements are well-known, it is still unclear whether or not lateral head turns would indeed activate the postural control system to maintain balance. This study aimed to examine the kinetic parameters in freely standing intact cats during head movements in order to further answer the above question.

**Methods:**

Six intact cats were trained to stand, unrestrained on a force plate and perform voluntary head movements to the left and right positions in response to visual cues. Each trial was divided into two phases, quiet standing with the cat’s head maintaining a straight forward and lateral head position after voluntary head movements. Kinetic parameters including peak pressure and contact area under each limb as well as center of pressure (COP) displacements of the whole body were measured.

**Results:**

Compared to the neutral head position, peak pressure and contact area of the left head position were significantly smaller for the left forelimb while greatly larger for the right forelimb. An exact opposite case of peak pressure and contact area in the forelimbs was found between the right and neutral head positions. In addition, the COP displacements altered oppositely to the head movements, and presented a significantly right shift in the left position and a significantly left shift in the right position.

**Conclusion:**

These results demonstrate that the lateral displacement of the head in standing intact cats does activate the postural adjustment to maintain balance, which is consistent with the concept that vestibular input can contribute to postural balance during voluntary head turns.

## Introduction

Postural control, such as standing still, is a complicated motor task that involves nearly all the body segments (Macpherson, Lywood & Van Eyken, 1987). It involves the integration of several sensory inputs, such as visual, vestibular and somatosensory (cutaneous and proprioceptive) inputs to maintain balance, and assesses each input on the basis of the condition and previous experience with the task (Allum & Pfaltz, 1985; Horak, Nashner & Diener, 1990; Inglis & Macpherson, 1995; Macpherson, Lywood & Van Eyken, 1987; Stapley et al., 2006). Combining these inputs can provide a full view of body orientation and dynamics within a particular condition, from which the different postural control responses for maintaining balance can be further adjusted (Stapley et al., 2006).

Previous studies have demonstrated that the vestibular afferent information is very important for many motor tasks. Interestingly, one of the most significant features of bilateral vestibular damage is that it only produces acutely profound effects on certain motor behaviors, especially on voluntary head movements (Keshner, 1994; Macpherson & Inglis, 1993; Stapley et al., 2006). For instance, following bilateral removal of the vestibular apparatus, cats respond normally to translation in the horizontal plane, but they lose balance and fall to their ipsilateral side when making lateral head movements during standing or locomotion (Inglis & Macpherson, 1995; Marchand, Amblard & Cremieux, 1988; Thomson et al., 1991). Likewise, people with vestibular loss can also keep postural balance during translation of the support surface, but even patients with bilateral deficit of vestibular function who have fully recovered, still become unstable when turning their head laterally during walking or when performing certain motor tasks (Allum, Honegger & Schicks, 1993; Allum & Pfaltz, 1985; Horak, Nashner & Diener, 1990; Lacour & Borel, 1993).

While these cases in which humans and cats with bilateral vestibular loss have difficulty with postural balance are well-documented, the potential causes remain poorly understood.

One of the most mainstream explanations for the mechanism underlying poor postural balance in individuals with bilateral vestibular loss is that vestibular and neck afferent inputs are both fundamental to exactly calculate the position of trunk in space when the head is turning laterally, thus the loss of vestibular or neck afferent signals would cause humans and cats to fail in maintaining postural balance (Mergner, Huber & Becker, 1997; Stapley et al., 2006). Previous studies have almost exclusively investigated the effect of bilateral vestibular loss on balance during lateral head movements (Keshner, 1994; Stapley et al., 2006). Nevertheless, it must be fully confirmed that whether voluntary head movements would indeed activate the postural control system to maintain balance before the above explanation can be further adopted. To date, few studies have been done to examine this. In addition, there is no study that has focused on the kinetic parameters of intact cats during voluntary head movements.

Therefore, the purpose of this study was to re-investigate whether lateral head movements would consequently activate the postural control system to maintain balance by comparing the kinetic parameters (peak pressure, contact area and center of pressure (COP)) of standing intact cats between the left, right and neutral positions of the head. We hypothesized that cats would present altered kinetic characteristics during lateral head movements, and these differences may generate the signals that the body requires to keep postural balance.

## Methods

### Animals

Six clinically-intact Chinese garden cats, ranging in mass from 2.5 to 2.8 kg, were recruited for the study. All cats were ensured clinically intact based on a complete physical examination performed by the same veterinarian. This study was approved by the Animal Care and Use Ethics Committee of Ningbo University (RAGH20190213). Informed client consent forms were obtained from the owners before the test.

### Experimental equipment

**Figure 1:**
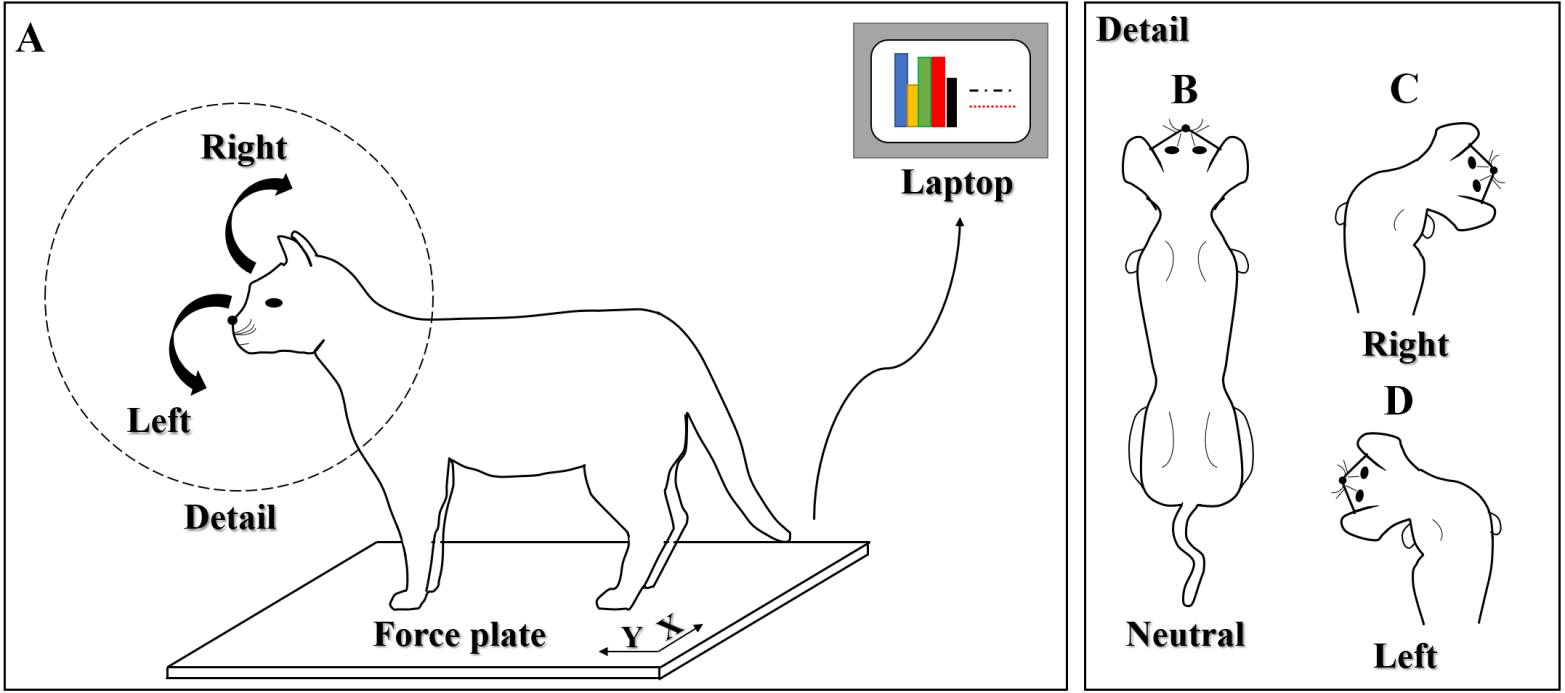
A schematic view of voluntary head movements in standing cats. (A) Overall view; (B) neutral head position; (C) right head position; (D) left head position.

The kinetic data were collected by a 0.395 m × 0.24 m force plate (Emed^®^-m pedography platforms, Novel GmbH, Germany) containing 3,792 sensors (four sensors per cm^2^) (Fig. 1). The force plate was connected to a laptop computer and collected data were analysed using designated software (Novel database essential 13.3.42, Novel GmbH, Germany) provided by the manufacturer. The data acquisition parameter was set to a frequency of 50 Hz. Prior to data acquisition, calibration of the force plate was performed based on the manufacturer’s instructions.

### Experimental design

This test was conducted in a quiet room with only the researchers and the cat owner (s) present. Each cat was trained using food and verbal reinforcement to stand on the force plate with their body mass equally distributed on each side. They were also trained to perform voluntary head turns to the left or right position in response to visual cues (Fig. 1). The cat owner squatted directly in front of the cat without disturbing it, and then moved a toy or something that the cat is familiar with to its left or right to elicit rapid head turns. Trials were excluded if: (1) the cat moved before the visual cues; (2) the cat’s body mass was not equally distributed on each side before the cue; (3) the cat lifted its limbs off the plate during quiet standing or lateral head movements. Five valid trials for each direction of head movements were obtained from each cat for further analysis.

Each trial consisted of two phases: (1) quiet standing with cat’s head maintaining a straight forward position; (2) lateral head positions after voluntary head movements. The plate was divided into four regions according to cat’s body structure, including left forelimb (LF), right forelimb (RF), left hindlimb (LH) and right hindlimb (RH). Kinetic variables including peak pressure (peak pressure distributed to each region measured from the force plate) and contact area (the contact area of plantar surface with the force plate) under each limb, as well as the COP displacement of the whole body at both phases, which was resolved into the medial-lateral displacement (COPx) and anterior-posterior displacement (COPy), were processed for further analysis.

### Statistical analysis

Statistical analysis was performed using SPSS version 17.0 software (SPSS Inc., Chicago, IL, USA). Initial Shapiro–Wilk tests validated that the data were normally distributed. An independent sample *T*-test was used to identify statistically significant differences of peak pressure, contact area, COPx and COPy between the left, right and neutral positions of the head in standing cats. All data are presented as means ± SD. Statistical significance was set at *p* < 0.05.

## Results

### Peak pressure

**Figure 2:**
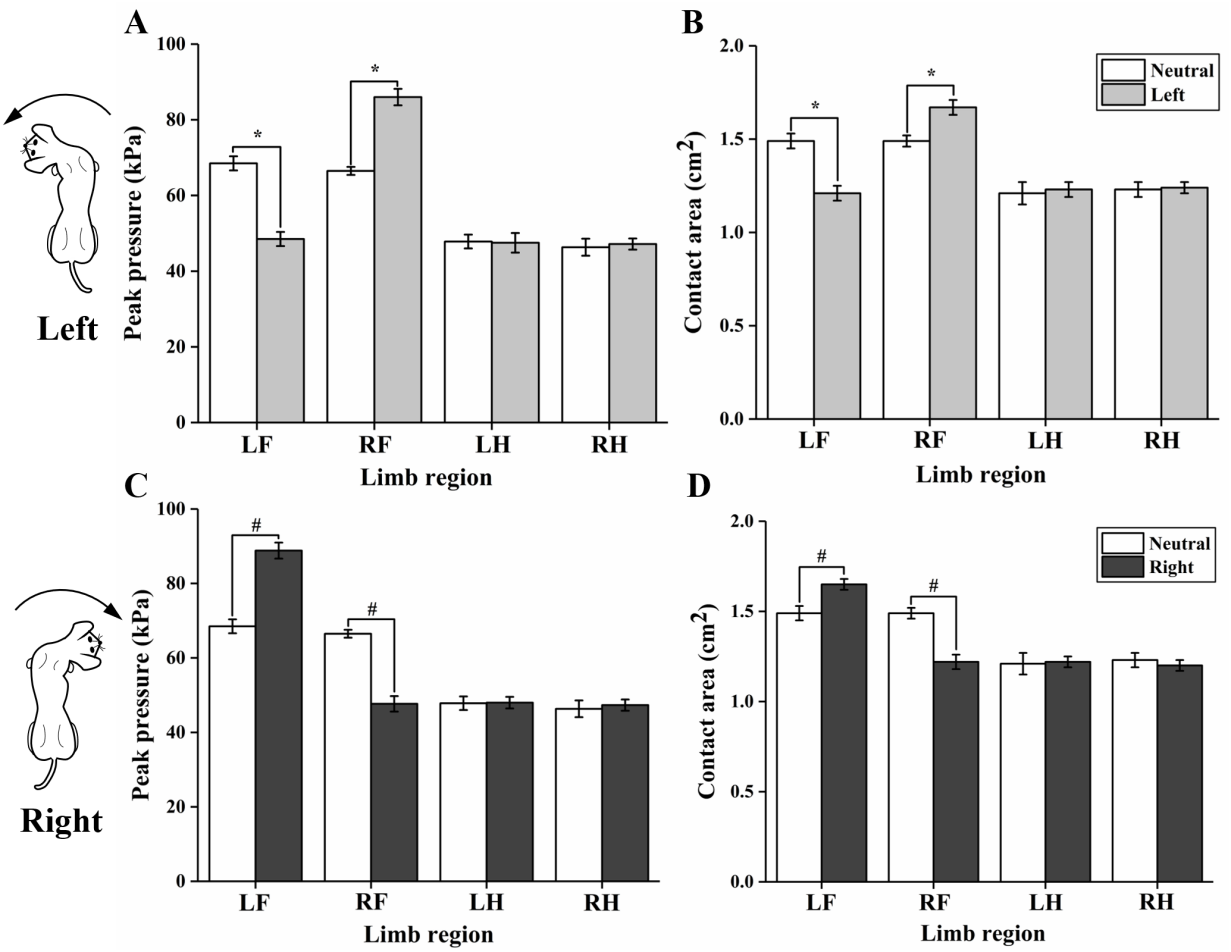
Comparisons of peak pressure and contact area between the left, right, and neutral head positions during voluntary head movements. Left forelimb, LF; right forelimb, RF; left hindlimb, LH; right hindlimb, RH. (A) Comparisons of peak pressure between the left and neutral head positions; (B) comparisons of contact area between the left and neutral head positions; (C) comparisons of peak pressure between the right and neutral head positions; (D) comparisons of contact area between the right and neutral head positions. An asterisk indicates the significant difference between left and neutral head positions; “#” means the significant difference between right and neutral head positions.

As shown in Table 1 and Fig. 2, peak pressure in LF of the left head position was significantly smaller than that of the neutral head position (*p* < 0.001), while peak pressure in RF was greater than that of the neutral head position (*p* < 0.001). However, an opposite case of peak pressure was observed between the right and neutral head positions, peak pressure in LF of the right head position was significantly larger than that of the neutral head position (*p* < 0.001), but was smaller in RF (*p* <  0.001). There was no significant difference in peak pressure between the left, right, and neutral head positions in hindlimbs (*p* > 0.05).

**Table 1:** Comparison of peak pressure, contact area of each limb and COP displacements of whole body between the left, right, and neutral positions during voluntary head movements.

Variables	Neutral position	Left position	*P* value	Power	Neutral position	Right position	*P* value	Power
	Mean ± SD	Mean ± SD			Mean ± SD	Mean ± SD		
Peak pressure (kPa)								
Left forelimb	68.50 ± 1.87	48.50 ± 1.87	0.000	1.000	68.50 ± 1.87	88.83 ± 2.14	0.000	1.000
Right forelimb	66.50 ± 1.05	86.00 ± 2.19	0.000	1.000	66.50 ± 1.05	47.67 ± 2.07	0.000	1.000
Left hindlimb	47.83 ± 1.83	47.50 ± 2.59	0.802	0.079	47.83 ± 1.83	48.00 ± 1.55	0.868	0.069
Right hindlimb	46.33 ± 2.25	47.17 ± 1.47	0.465	0.176	46.33 ± 2.25	47.33 ± 1.51	0.387	0.211
Contact area (cm^2^)								
Left forelimb	1.49 ± 0.04	1.21 ± 0.04	0.000	1.000	1.49 ± 0.04	1.65 ± 0.03	0.000	1.000
Right forelimb	1.49 ± 0.03	1.67 ± 0.04	0.000	1.000	1.49 ± 0.03	1.22 ± 0.04	0.000	1.000
Left hindlimb	1.21 ± 0.06	1.23 ± 0.04	0.499	0.156	1.21 ± 0.06	1.22 ± 0.03	0.631	0.096
Right hindlimb	1.23 ± 0.04	1.24 ± 0.03	0.541	0.118	1.23 ± 0.04	1.20 ± 0.03	0.235	0.392
COP displacement (cm)								
COPx	10.64 ± 0.24	11.75 ± 0.20	0.000	1.000	10.64 ± 0.24	9.69 ± 0.09	0.000	1.000
COPy	18.03 ± 0.20	17.72 ± 0.31	0.070	0.607	18.03 ± 0.20	18.01 ± 0.17	0.857	0.071

### Contact area

The average of cats’ footprints in diameter is 2.1 ± 0.2 cm, both in the fore- and hind limbs. The contact area of the left, right, and neutral head positions during lateral head movements are shown in Table 1 and Fig. 2. Contact area of the left head position was significantly smaller in LF (*p*  <  0.001), but larger in RF (*p* < 0.001) when compared with that of the neutral head position. However, an opposite case of contact area was observed between the right and neutral head positions which is similar to the trend of peak pressure. The right head position showed larger contact area in LF (*p* < 0.001) and smaller contact area in RF (*p* < 0.001) than that of the neutral head position. No significant difference was found between the left, right, and neutral head positions in hindlimbs (*p*  >  0.05).

### Centre of pressure

**Figure 3:**
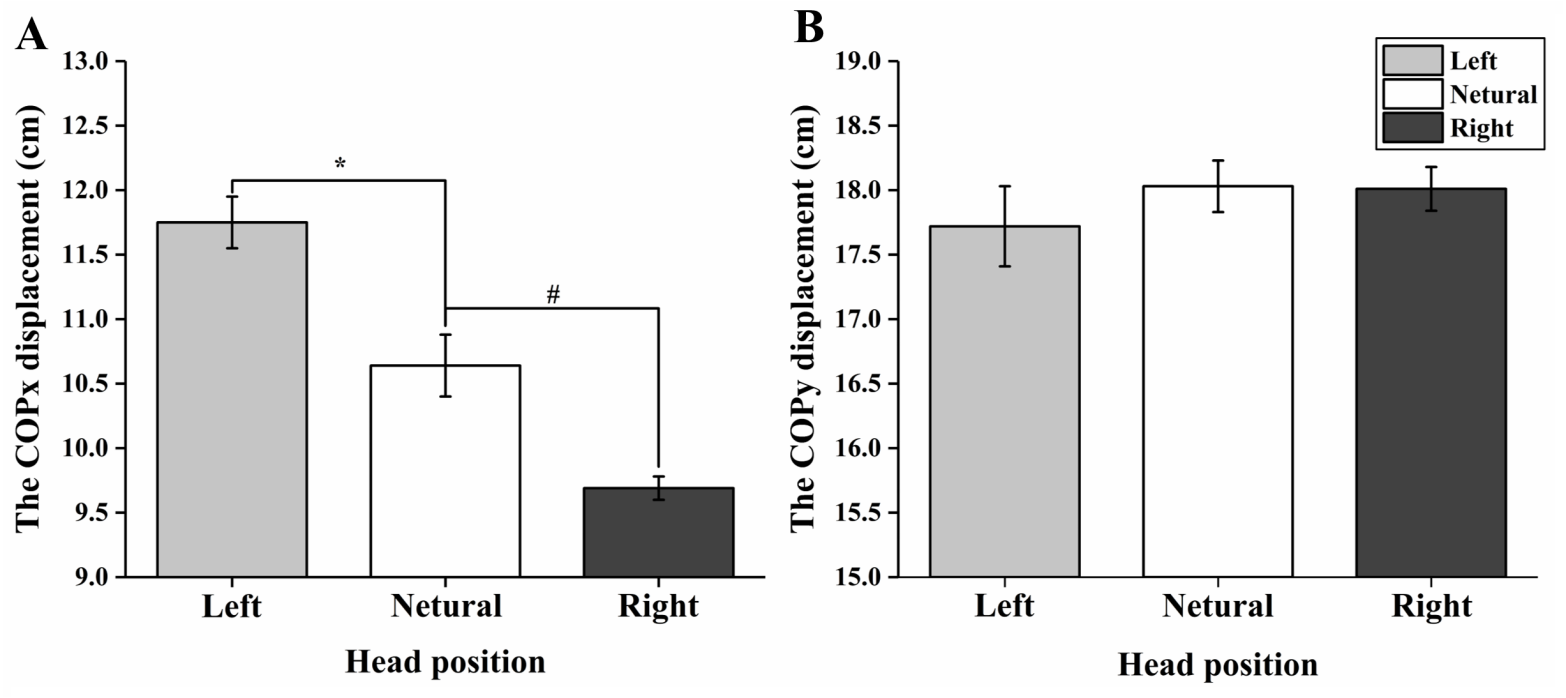
Comparison of COP displacements between the left, right, and neutral positions during voluntary head movements. (A) Comparison of COPx displacement between the left, right, and neutral head positions; (B) Comparison of COPy displacement between the left, right, and neutral head positions. An asterisk indicates the significant difference between left and neutral head positions; “#” means the significant difference between right and neutral head positions.

Statistical analysis revealed that the differences in COPx between the left, right, and neutral head positions were significant, while the differences did not reach to a significant level when comparisons were made for COPy (Table 1 and Fig. 3). The COP displacement presented a significantly right shift at the left head position (*p* < 0.001) and a significantly left shift at the right head position (*p* < 0.001). However, no significantly anterior-posterior shift was observed during voluntary head movements (*p* >  0.05).

## Discussion

This study aimed to compare the kinetic parameters of standing cats between the left, right and neutral positions of the head in order to examine whether the lateral head turns would indeed activate postural control system to maintain balance. Consistent with our hypothesis, results of the present study indicated that peak pressure and contact area of contralateral forelimb in the left and right head positions are significantly larger than in the neutral position, while peak pressure and contact area of ipsilateral forelimb reduced greatly during voluntary head movements. Additionally, the center of pressure (COP) shifted significantly in medial-lateral direction during voluntary head movements. The average of contact area seems to be smaller comparing to previous study, as footprints of the domestic cats are approximately three cm in diameter both in the fore- and hind limbs (Douglas de Carvalho et al., 2015). However, the breed of cats that were recruited in this study is Chinese garden cat, and their footprints are highly smaller than that of the domestic cat mentioned in the above study.

During the lateral head movements, the left and right head positions were found to be symmetric as expected in altered kinetic parameters. Therefore, for the purpose of clarity, only the comparisons between the left and neutral head positions will be discussed in this study. In general, the weight of the cat’s head is about 13% of the total body mass (Hoy & Zernicke, 1985). Therefore, from a mechanics perspective, the contralateral forelimb should exhibit increased load while the ipsilateral forelimb should demonstrate a reduced load to keep weight balance during left head turn. Indeed, in our study we observed a larger peak pressure and contact area in the right forelimb and smaller peak pressure and contact area in the left forelimb when the head turned to the left. Moreover, this finding is consistent with the significant right shift of the COPx during left head movement (Fig. 3). These changes may be indicating, that the cause and reasons why voluntary head movements activate postural adjustment systems to maintain balance are partially explained.

There is a chance that the changes in cat posture are associated with anticipatory postural adjustments before a voluntary head rotation, which means the postural changes precede the movement onset rather than respond to perturbation (Massion, 1984). However, it has been demonstrated that anticipatory postural adjustments associated with voluntary movements were attenuated or absent when the posture was unstable as well as when it was very stable (Aruin, Forrest & Latash, 1998). In addition, at the early phase of the left head turn, most of the lesioned cats did show similarities with the intact cats, and then their right forelimbs would generate a greater thrust that was able to push the whole body to the left side and lead to falling (Stapley et al., 2006). These explanations would support the point from another perspective that it is the voluntary head movements that result in kinetic changes of cats rather than anticipatory postural adjustments.

After bilateral vestibular removal, cats would lose balance and fall to their ipsilateral side when performing voluntary head movements during standing or locomotion (Inglis & Macpherson, 1995; Marchand, Amblard & Cremieux, 1988; Thomson et al., 1991). We think that the loss of vestibular afferent inputs disrupts the postural control system, such that lesioned cats cannot balance the force loaded on the right forelimbs to correctly adjust the trunk position. Furthermore, when the lesioned cats turn their head left, the lack of vestibular afferent information may also misdirect the nervous system by making it wrongly perceive that the trunk is leaning to the right side, which would improperly activate the postural control system and then lead to falling (Stapley et al., 2006).

One potential limitation of this study should be noted. We only used the kinetic parameters to examine whether there are some effects of voluntary head movements on postural balance. However, it would be much clearer if we further investigated the electromyographic activities of cat’s limbs as this could outline show the pattern that these limb muscles follow during lateral head turns.

## Conclusions

This study compared the kinetic parameters of the standing cats between the left, right and neutral positions of the head during voluntary head movements. Based on the findings of this study, it was demonstrated that lateral head movements would activate the postural adjustment system to maintain balance during these movements. However, further research studies to explore the internal mechanism of this change are much needed.

##  Supplemental Information

10.7717/peerj.8186/supp-1 Supplemental Information 1 Raw data of the peak pressure during voluntary head movements exported from force plate applied for data analyses and preparation for the detailed investigation shown in Fig. 2 and Table 1

10.7717/peerj.8186/supp-2 Supplemental Information 2 Raw data of the contact area during voluntary head movements exported from force plate applied for data analyses and preparation for the detailed investigation shown in Fig. 2 and Table 1

10.7717/peerj.8186/supp-3 Supplemental Information 3 Raw data of the center of pressure during voluntary head movements exported from force plate applied for data analyses and preparation for the detailed investigation shown in Fig. 3 and Table 1

## Additional Information and Declarations

### Data Availability

The following information was supplied regarding data availability: Raw data is available in the Supplementary Files.
